# Aqueous Extract of *Origanum majorana *at Low Temperature (0°C) Promotes Mitochondrial Fusion and Contributes to Induced Apoptosis in Human Breast Cancer Cells

**DOI:** 10.31557/APJCP.2021.22.9.2959

**Published:** 2021-09

**Authors:** Asma Algebaly, Qwait Algabbani, Wedad Refaiea Al-Otaibi, Amani Mohammed Alotaibi, Fatimah Gh. Albani, Ibtesam Sanad Alanazi, Gadah Albasher, Abdul Qader Saeed Alqahtani, Wedad Saeed Al-Qahtani

**Affiliations:** 1 *Department of Biology, College of Sciences, Princess Nourah Bint Abdulrahman University, Riyadh, Saudi Arabia. *; 2 *Department of Biology, College of Sciences and Humanities, Prince Sattam Bin Abdulaziz University, Al-Kharj, Saudi Arabia. *; 3 *King Saud Medical City, Riyadh, Saudi Arabia. *; 4 *Department of Biology, Faculty of Sciences, University of Hafr Al-Batin, Saudi Arabia. *; 5 *Department of Zoology, College of Science, King Saud University, Riyadh, Saudi Arabia. *; 6 *Royal commission hospital, Pharmacy Department, Saudi Arabia. *; 7 *Department of Forensic Sciences, College of Criminal Justice, Naif Arab University for Security Sciences, Riyadh, Saudi Arabia. *

**Keywords:** Origanum majorana, human breast cancer cells, MCF7, aqueous extract at low temperature (0°C/6 hours)

## Abstract

**Objective:**

Marjoram plants have varied pharmacological properties because they contain antioxidants. In the present study, the effect of Origanum majorana, gathered from Abha, Saudi Arabia, was evaluated on the growth of MCF7 breast cancer cells.

**Methods:**

Fresh aerial parts from *Origanum majorana* were extracted at a low temperature (0 ^O^C/6 hours). MCF7 human breast cancer cells were then treated with 4 separate fluctuated concentrations of 0, 50, 150, 200 and 350 µg/mL for 24 and 48 hours.

**Results:**

The findings showed that *Origanum majorana* aqueous extract contained absolute phenolic content (TPC) of 58.24 mg equivalent/g DW, and the complete flavonoid content (TFC) of 35.31 mg GAE equivalent/g DW. The endurance of MCF7 cells after incubation with aqueous extract diminished, indicating that *Origanum majorana* is tumour cell selective. *Origanum majorana* extract increased the mRNA expression of apoptotic genes in MCF7. The majorana aqueous extract expanded the activity of Caspase-7 action specifically at higher concentrations of 150, 200, and 350 µg/ml. Our findings indicate that Origanum majorana could induce apoptosis of human breast cancer cells.

**Conclusion:**

The aqueous *Origanum majorana* extracted at low temperature (0 °C/6 hours) can be as a anti-cancer treatment agent if further studies wanrents support our result.

## Introduction

Origanum majorana (*O. majorana*), an herbaceous and perennial plant, is native to countries in southern Europe and the Mediterranean region (Tucker and Maciarello, 1994). Marjoram plants vary in their pharmacological properties as a result of varying concentrations of antioxidants contained within them (Leeja and Thoppil, 2007; Rasool, 2012). The diverse chemical compositions and aromas of marjoram plants have made them useful for flavouring food products and alcoholic beverages. Industrial researchers use whole plants and plant parts rich in bioactive phytochemicals in the preparation of foods and medicines (Leeja and Thoppil, 2007). While a significant number of synthetic antioxidant-rich foods and food supplements are sold in markets, plants and plant components that contain natural antioxidants are preferred over synthetic supplements due to fewer side effects (El-Ashmawy et al., 2007; Rasool, 2012).

*O. majorana* plants are traditionally used for their medicinal properties and has been used as an antibacterial, antithrombin, and antihyperglycemic treatment (Sari et al., 2006). These plants also contain vital antioxidants, such as triterpenoids and flavonoids (Cipak et al., 2006; Gad et al., 2020). Marjoram plants contain phenolic glycosides (arbutin, methyl arbutin, vitexin, orientin, and thymonin), tannins, phenolic terpenoids (thymol and carvacrol), hydroquinone, flavonoids (apigenin, diosmetin and luteolin) and triterpenoids (oleanolic acid and ursolic acid) (Attoub et al., 2018; Niture et al., 2006). The ability of natural antioxidants to scavenge free radicals and protect cells from comorbidities such as cancer has attracted extensive research interest. Extracts from the marjoram plants both aqueous and ethanolic are critical in the prevention of cancer and oncogenic mutations (Amor et al., 2019; Niture et al., 2006). *O. majorana* is widely used in folk medicine; for example, marjoram tea (extracted from its leaves) is widely used to mitigate the symptoms of stomach pain, hay fever, dizziness, sinus congestion, cold, indigestion, cough, asthma, headache, and nervous disorders (Leeja and Thoppil, 2007; Richter and Schellenberg, 2007; Vági et al., 2005). Nevertheless, no report to date is available on the bioactive components of *O. majorana* aqueous extract at low temperature (0°C/6 h) acquired from species developed in Abha, Saudi Arabia, just as their pharmacological activities on the fission of the mitochondria contributed to apoptosis in malignant human breast cells. Hence, the present investigation aimed to analyse the chemical composition of *O. majorana* aqueous extract that was extracted at low temperature (0°C/6 h) and to explain the effect of this chemical composition of *O. majorana* aqueous extract on a MCF7 cell line.

## Materials and Methods

*Preparation of aqueous suspension and plant extracts*

**Figure 1:**
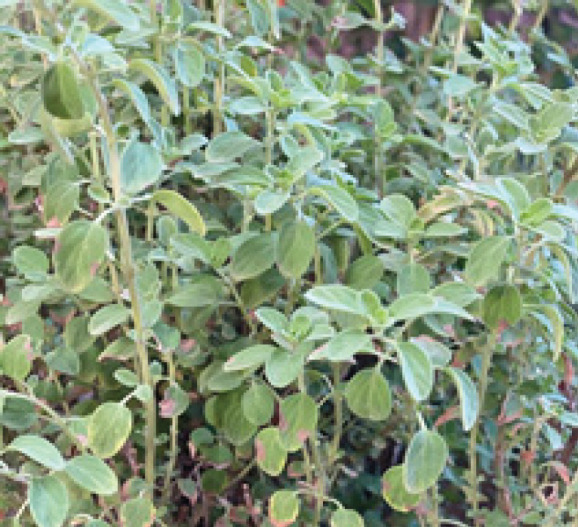
*Origanum majorana (O. majorana) *Plant was Uutilized in the Current Study, Abha, Saudi Arabia

Aerial parts from *O. majorana* were collected from the city of Abha in southern Saudi Arabia and identified by Dr. AbdulQader S. Alqahtani (Pharmacy Department, Royal Commission Hospital, Saudi Arabia). The voucher specimen was coded (#32651) and stored in the Department of Biology of Princess Nourah bint Abdulrahman University in Riyadh, Saudi Arabia (Figure 1). The plants (500 g) were cleaned under running water, squashed, and blended in deionized water (1:20 w/v) prior to extraction at 0^o^C for 6 h. The rough extract was centrifuged at 3000 rpm for approximately 15 min before the use of a vacuum freeze dryer (Model FDF 0350, Korea) to lyophilize the plant extract. The resulting gooey powder was then divided into correct stock arrangement volume of centralization (50 mg/ml). The extract yield was noted to be 14.3% per 100 g. The extract was then stored as stock at 4°C. Table 1 shows the plant descriptions used in this study. 

*Phytochemical analysis*

Initial phytochemical analysis employed the standardised techniques outlined by Wagner et al., (2011) to detect the components of essential oils (saponins, alkaloids, and terpenoids).

*Assessment of Anthocyanins and Anthraquinones*

*O. majorana* aqueous extract was screened for carried anthocyanins and anthraquinones according to standard methods reported by in Abduljalil et al., (2018).

*Analysis of Total Phenolic (TPC), Flavonoid Contents (TFC)*

Folin–Ciocalteu reagent was used to determine the total phenolic content (TPC) as outlined by Chandra and Gonzalez (2004). The absorbance was recorded at 735 nm against a blank. The amount of TPC was calculated as gallic acid equivalent from the standard calibration curve of gallic acid (y = 0.0084x + 0.0304, R^2^ = 0.9979) and converted to mg gallic acid equivalents (GAE)/g DW basis (Woisky and Salatino, 1998).

*Condensed tannin content analysis (CTC)*

Julkunen-Titto method (Julkunen-Tiitto, 1985), with slight variation as outlined by Salar and Purewal (2016), was used to determine the condensed tannin content. The readings of the absorbance against the blank were performed at 500 nm. Catechin was utilized to prepare the standard curve. The results of the measurement were converted to mg catechin equivalent/g dry weight basis (mg CE/g DW). 

*Cell culture and treatment conditions*

Human MCF7 cells purchased from the American Type Culture Collection (ATCC, Manassas, VA, USA) were stored in DMEM/Ham’s F-12 (1:1 v/v) medium that had been enhanced with 400 µg/ml hydrocortisone, streptomycin (0.1 mg/ml), 100 ml/L FBS, 1.5 g/L sodium bicarbonate, and 10 ml/L penicillin. Cells were seeded at a concentration of 1×10^5^ cells/well or 1×10^6 ^cells/well in 96- and 25-well tissue culture plates independently in the humified hatchery. Thereafter, aqueous extract from the aerial part of the *O. majorana* was placed in culture media, treated, and combined with four independent concentrations of 0, 50, 150, 200, and 350 µg/ml for 24 and 48 h.

*Direct cell counting with a haemocytometer*

Following treatment, a pipette was used to mix the cells with 20 μl PBS per well in 96-well plates. A single drop was placed in the haemocytometer from each of the 96-well plates. Living cells were observed to be round and transparent, whereas dead cells were shrunken and dense. The formula below was used to compute the percentage of cell viability.

%cellviability=Absorbance of treatedandorexposedviable cellsAbsorbance of (untreated and unexposed) viable cells

*Glucose Uptake Measurement*

MCF7 cells were treated with 2-[N-(7-nitrobenz-2-oxa-1,3-diazol-4-yl) amino]-2-deoxy-D-glucose (2-NBDG) for 30 min after first being treated for 24 h with *O. majorana* extract at concentrations of 0, 50, 150, 200, or 350 µg/ml. Flow cytometry (Becton-Dickinson, San Jose, CA) was used to determine glucose uptake in MCF7 human breast cancer cells. 

*Production of reactive oxygen species*

Fluorometry was used to examine the production of subcellular ROS via estimation of ROS of a DCF-DA (non-fluorescent 2ʹ,7ʹ-dichlorofluorescein diacetate) oxidation to a fluorescent metabolite dichlorofluorescein (DCF) via mitochondrial ROS after 24-h treatment with *O. majorana* extract at concentrations of 0, 50, 150, 200, or 350 µg/ml. This estimation was done with slight variations (Grbović et al., 2013).

*RNA Extraction and cDNA Synthesis*

The cells were treated by Origanum majorana extract 0, 50, 150, 200, and 350 µg/ml for 24 and 48 hours and collected as pellets in triplicated per tested concentration. The Invitrogen-TRIzol reagent was used to disintegrate all out RNA as per the manufacturer’s guidelines and followed by the estimation of the absorbency at 260 nm. Thereafter, the synthesizing of the cDNA-strand was done using the High-Cpacity cDNA reverse transcription kit (Applied Biosystems) based on the manufacturer’s instructions and by (Zordoky et al., 2008).

*Measurement of mRNA Expressions by Real-Time Polymerase Chain Reactions (RT-PCR)*

Table 2 shows primers acquired from (Invitrogen, USA) and used in the present examination. As per the manufacturing directions, Real-time PCR was conducted using an ABI 7500 PRISM Kit (Applied Biosystems) to run the controls and the samples in triplicates. A brief centrifugal step was performed, and the PCR plate was subjected to 40 cycles: (i) PCR initiation at 95°C for 5 min, (ii) denaturation at 95°C for 5 sec, and (iii) annealing/augmentation at 60°C for 10 sec. The relative quality expression (i.e., ΔΔCT) strategy, as earlier outlined, was used to analyse RT-PCR data (Zordoky et al., 2008). GAPDH was used as a reference gene.

*Determination of Caspase-7 Activity*

Using BioVision (Mountain View, California, USA) CaspACE testing framework and following the manufacturer’s instructions, the activity of caspase-7 was estimated calorimetrically. The MCF7 cells were plated onto 12-welled cell culture plates and treated for 24 h with *O. majorana* aqueous extracts at concentrations of 0, 50, 150, 200, and 350 µg/ml. Thereafter, trypsinization was used to collect the MCF7 cells, which were then suspended in cold cell lysis cushions that had been treated with ice for 10 min. The suspension was then centrifuged at 10,000×g at 4°C and placed in a crisp cylinder for storage at −20°C. To estimate caspase-7 actions, approximately 30 μg of extracted protein from the control cells and tested cells were incubated in 200 μM compound clear-cut, colorimetric caspase-7 substrate I (Ac-DEVD-pNA), Acetyl-Asp-Glu-Val-Asp p-nitroanilide, for 2 h at 37°C. Caspase-7 activity was estimated by evaluating the absorbance at 405 nm using a perused plate (Bio-Tek Instruments, Winooski, VT). 

*Mitochondrial fission*

Following the treatment of MCF7 cells by *O. majorana* extracts at concentrations of 0, 50, 150, 200, and 350 µg/ml for 24 h, the cells were treated with 250 nM MitoTracker Deep Red FM (Invitrogen) in free culture for approximately 30 min. Using PBC, the cells were then washed, and Hoechst 33342 was applied for 10 min to re-stain the nuclei. Mitochondrial morphology within the cells was then observed using a Zeiss LSM700 confocal microscope.

*Analysis of Statistics*

The SigmaStat programming software v. 3.5 (Systat Software, San Jose, CA, USA) was used for statistical analyses. One-way ANOVA was used to assess glucose uptake data, ROS production, caspase-7 mRNA expression, and RNA synthesis inhibitor Act-D. Two-way ANOVA was used to assess cell viability and mRNA expression levels of genes related to oxidative stress apoptosis c the control of triplicate experiments. The results of the quantitative outcomes were expressed as means and standard deviations. P values below 0.05 were deemed statistically significant.

## Results

*Phytochemical Qualitative Analysis*

Table 3 shows the results of the subjective phytochemical investigation of *O. majorana* aqueous extracts. The findings reveal that the aqueous extract contains essential oils, glycosides, tannins, gums, mucilage, flavonoids, amino acids, and alkaloids, while terpenoids are absent. In addition, weak nearness of steroids in the *O. majorana* aqueous extract was observed.

*Phenolic and Flavonoids Profile*

Table 4 defines both the absolute flavonoids and phenolic content of the Origanum majorana with their scores being 58.24 and 35.31 mg equivalent/g DW, respectively. 

*Impact of O. majorana aqueous extract on MCF7 Cell viability*

The MCF7 cells were treated for 24 and 48 h for the purpose of establishing the cells’ proficiency at hindering the growth and proliferation of cancer cells. Cells were treated at concentrations of 0, 50, 150, 200, and 350 µg/ml prior to measurement of cell reasonability and expansion via direct counting. 

**Figure 2:**
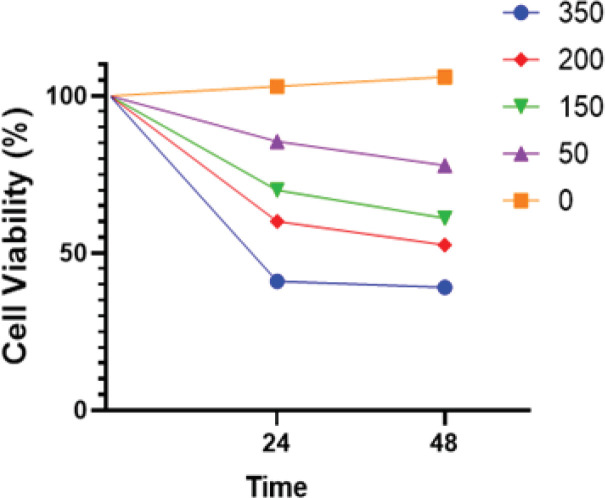
Impact of *O. majorana* Treatment on MCF7 Cell Viability and Growth. Values were presented as percentages (n = 5) relative to controls in three independent experiments

Figure 2 displays the decreasing endurance of MCF7 cells after incubation with *O. majorana* extracts relative to untreated MCF7 cells. The data further indicate that *O. majorana* may possess tumour-selective properties. The IC_50_ for the aqueous extract was approximately 350 µg/ml.

*Inhibition of glucose uptake and mitochondrial activity*

**Figure 3:**
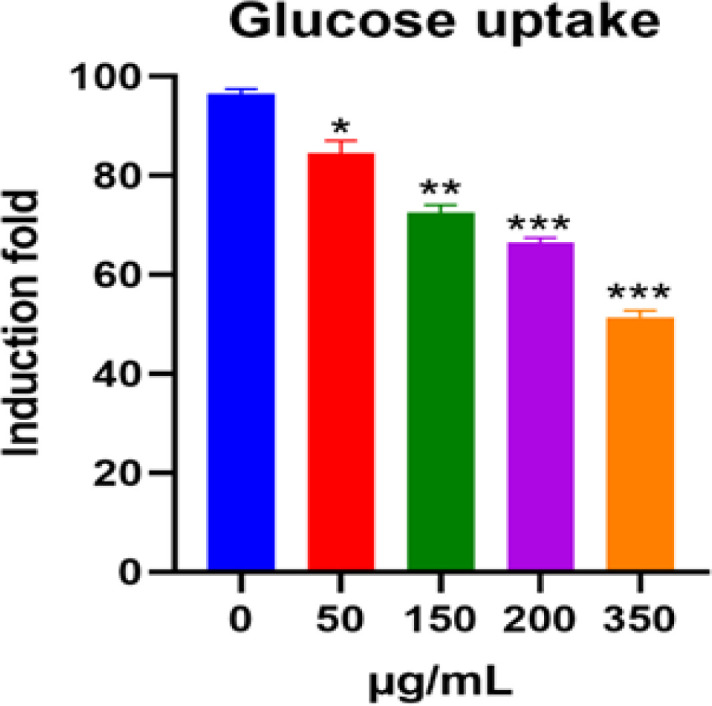
Suppression of Glucose Take-up in MCF7 Cells that were Treated by *O. majorana *Aqueous Extract for 24 hours. The glucose take-up was measured by flow cytometry. Data were represented as means ± SEM, (n = 5) of three tests. *P < 0.05, **P < 0.01 and ***P < 0.001 contrasted with control (0 µg/ml).

**Figure 4:**
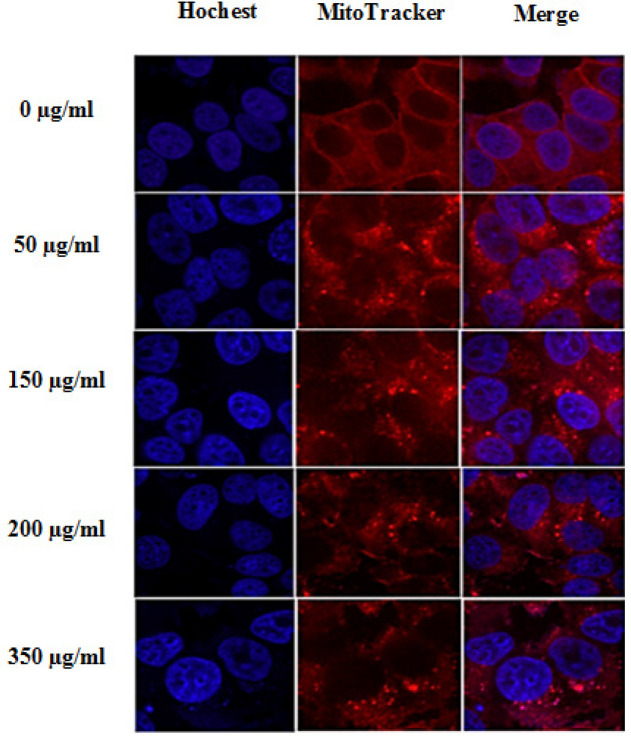
Photographs of Fluorescently Stained MCF7 Cells Indicating Pro-Apoptotic Status. Hoechst 33342 blue stain (first column) exhibits abnormal nuclei shapes, while MitoTracker Deep Red stain (second column) reveals mitochondrial fusion in the MCF7 cells treated with *O. majorana* aqueous extract (50, 150, 200, and 350 µg/ml) for 24 h in second, third, fourth and fifth raw, respectively. While the first raw show normal nucleic morphology with mitochondrial morphology of MCF7 cells (0 µg/ml). Images were captured by confocal microscopy

ATP production and cell viability are essential processes during glucose metabolism and cell growth. Hindering glucose absorption has been found to constrain cell growth. Our results indicate that 24-h treatment with aqueous extracts of *O. majorana* hindered glucose uptake (2-NBDG) in MCF7 cells in a dose-dependent manner. As shown in Figure 3, the results were 96.6% (control), 84.2% (50 µg/ml), 73.5% (150 µg/ml), 66.7% (200 µg/ml), and 51.8% (350 µg/ml) of *O. majorana* aqueous extract, respectively. As shown in Figure 4, treatment with *O. majorana* altered mitochondrial morphology in MCF7 cells, thereby making the mitochondrial splitting visible in cells treated with *O. majorana*.

*Effect of O. majorana aqueous extract on the Expression of Oxidative Stress Genes in MCF7 Cells*

**Figure 5:**
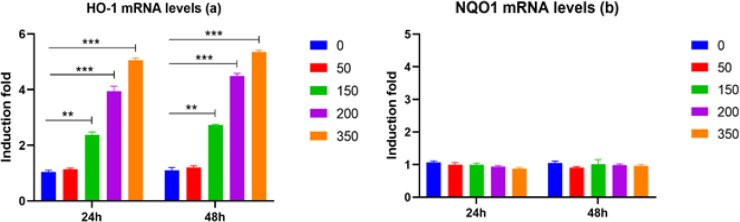
Effect of *O. majorana* Aqueous Extract on Oxidative Pressure Markers HO-1 (a) and NQO1 (b) mRNA Levels in MCF7 Cells Treated for 48 and 24 hours with Different Concentrations of *O. majorana* Aqueous Extract (0, 50, 150, 200, and 350 µg/ml). mRNA levels of HO-1 and NQO1 were measured utilizing RT-PCR and standardized to GAPDH housekeeping gene. Data were represented as means ± SEM (n = 5) of three tests. *P < 0.05, **P < 0.01 and ***P < 0.001 contrasted and untreated cells

**Figure 6:**
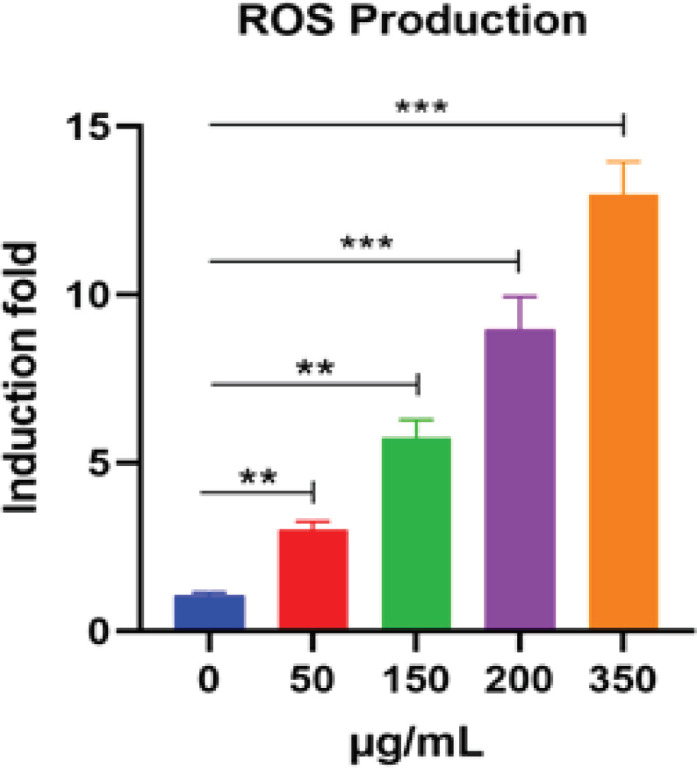
ROS Production in MCF7 Cells was Treated for 24 hours with Different Groupings of *O. majorana* Aqueous Extract (0, 50, 150, 200 and 350 µg/ml). From that point, cells were treated with DCF-DA (10 μM) for 1 hour. DCF values was estimated fluorometrically utilizing excitation/outflow frequencies of 484/535 nm. Data were recorded as means ± SEM, (n = 10) of three tests. *P < 0.05, **P < 0.01 and ***P < 0.001 contrasted with control (0 mg/ml).

The capacity of *O. majorana* aqueous extract to balance the pronouncement of oxidative pressure genes in MCF7 cells was assessed to examine the impact of increasing concentrations of *O. majorana* at 0°C/6 h on oxidative stress. MCF7 cells were treated with various concentrations of *O. majorana* for 24 and 48 h. Thereafter, DCF and RT-PCR were used to estimate ROS production and NQO1 and HO-1 mRNA expression. Figure 4 shows that treatment with *O. majorana* at concentrations of 150, 200, and 350 µg/ml significantly upregulated HO-1 mRNA expression in MCF7 cells in a dose-dependent manner (Figure 5a), which then induced HO-1 mRNA expression. However, *O. majorana* treatment did not alter NQO1 mRNA expression levels (Figure 5b), although it did increase ROS production at various concentrations in a dose-dependent manner (Figure 6). The 200 and 350 µg/ml of *O. majorana* aqueous extract generated the most significant acceptance of approximately 10- and 14-fold, respectively.

*Effect of O. majorana aqueous extract on the mRNA Expression Level of Apoptotic Genes in MCF7 Cells*

**Figure 7:**
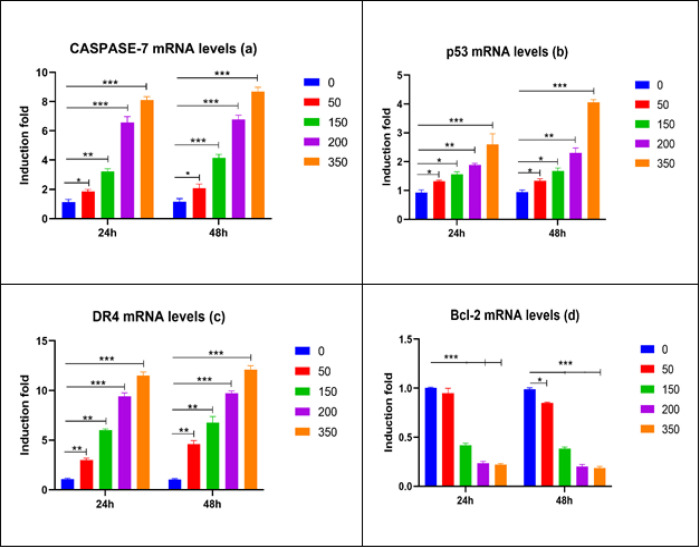
Impact of *O. majorana* Aqueous Extract on Apoptotic Markers Caspase-7 (a), p53 (b), DR4 (c), and BcL2 (d) mRNA levels in MCF7 Cells was Put under Treatment for 24 and 48 hours with Different Groupings of *O. majorana* Aqueous Extract (0, 50, 150, 200, and 350 µg/ml). mRNA levels of Caspase-7, p53, DR4, and BcL2 were evaluated utilizing RT-PCR and standardized to GAPDH housekeeping gene as depicted in techniques. Data were recorded as means ± SEM (n = 5) of three tests. *P < 0.05, **P < 0.01 and ***P < 0.001 contrasted with control (0 µg/ml).

We postulated that *O. majorana* aqueous extract was a determinant of the anti-apoptotic and apoptotic genes outflow in the investigation of the extract’s inhibitory effect on MCF7 cells activities such as expansion and development in apoptotic-interceded treatment. Consequently, treatment of the MCF7 cells was done for 24 and 48 hours while expanding concentrations of *O. majorana*, aerial parts aqueous extract from 0, 50, 150, 200 and 350 µg/m and as necessitated by the cell suitability. Figure 7 exhibits that *O. majorana* aqueous extract fundamentally activated p53, Caspase-7 and DR4 mRNA expression levels through a subordinate focus way (Figures 7 (a, b, c)). The most significant effect occurred at the highest elevations of 150, 200 and 350 µg/ml. Remarkably, of *O. majorana* aqueous extract (Figure 7(d)) caused a significant decrease in Bcl2 mRNA levels at the most important fixation tried (150, 200 and 350 µg/ml).

*Effect of O. majorana aqueous extract on Caspase-7 Activity in MCF7 Cells*

**Figure 8:**
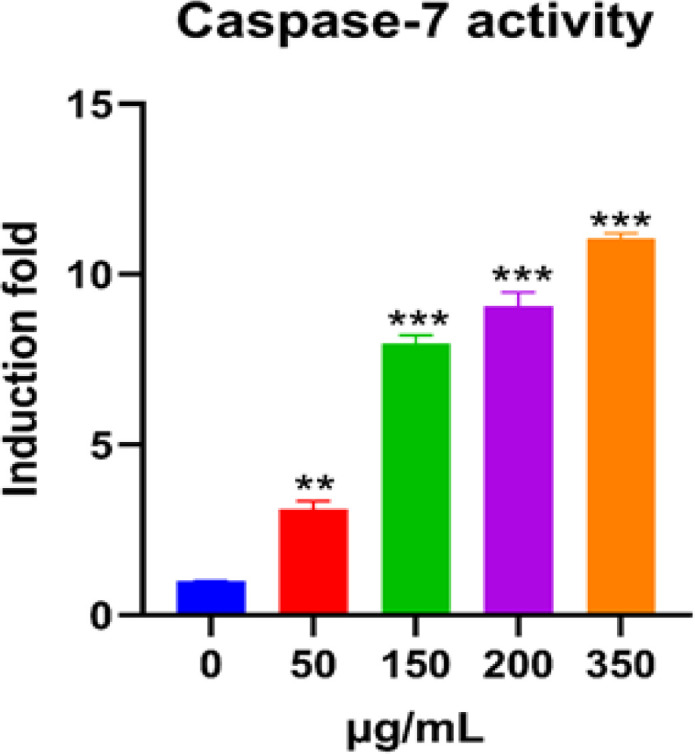
Impact of *O. majorana* Aqueous Extract on Apoptotic Caspase-7 Synergist Action in MCF7 Cells was Treated for 24 hours with Different Convergences of *O. majorana* Aqueous Extract (0, 50, 150, 200 and 350 µg/ml). From there on, Caspase-7 action was resolved calorimetrically utilizing the CaspACE examine framework bought from Biovision. Caspase-7 activity was evaluated by estimating absorbance at a frequency of 405 nm with a plate (Bio-Tek Instruments, Winooski, VT). Data were represented as means ± SEM (n = 5). *P < 0.05, **P < 0.01 and ***P < 0.001 contrasted with control (0 mg/ml).

The aqueous extract in MCF7 cells was changed into functional catalytic activities to inspect the effect of the *O. majorana* aqueous extract on Caspase-7 action. Consequently, MCF7 cells were treated for 24 hours, with increasing convergences of the aqueous extract. Thereafter, the Caspase-7 activities resolved using calorimetry by the use of the biovision kit in line with the methods. In Figure 8, the activity of the Caspase-7 action increased at higher concentrations, 150, 200, and 350 µg/ml by about 7, 8 and 12 folds, separately.

*Effect of the Transcription Inhibitor, Act-D, on the Induction of Caspase-7 mRNA by (O. majorana) aqueous extract in MCF7 Cells*

The investigation of whether the development in Caspase-7 mRNA by *O. majorana* aqueous extract in MCF7 cells is linked to an expansion in the de novo RNA union, MCF7 cells were treated for 24 hours with *O. majorana* aqueous extract while using the most elevated portion (350 µg/ml) in the nearness or nonappearance of 10 μg/ml actinomycin D (Act-D), which served as an RNA synthesis inhibitor. The assessment of the Caspase-7 mRNA was then done using RT-PCR. Where the *O. majorana* aqueous extract expands the measure of Caspase-7 mRNA via its de novo RNA synthesis under the set conditions, the substance of Caspase-7 mRNA would decline after the limitation of its RNA synthesis.

**Figure 9:**
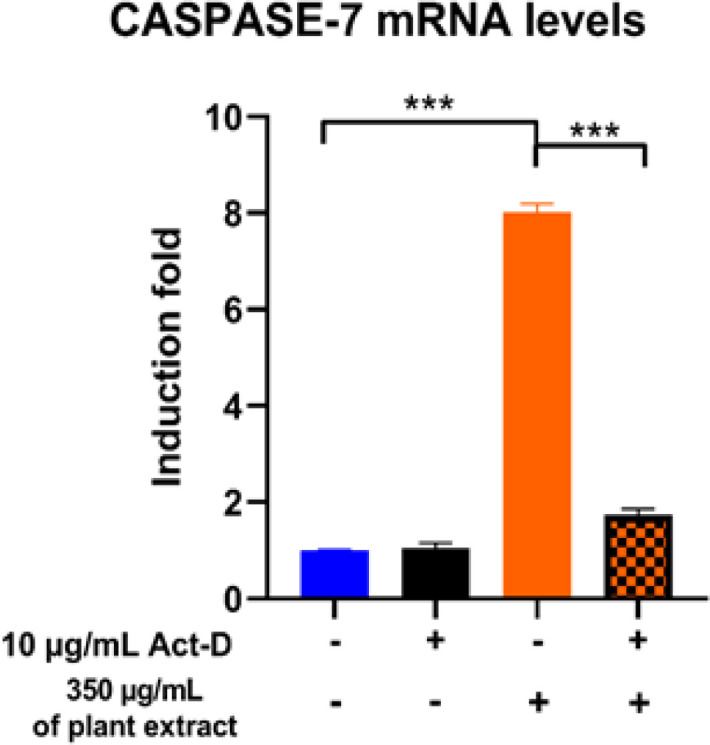
Impact of Act-D on the Activity of Caspase-7 by *O. majorana* Treatment in MCF7 Cells that were also Treated with 10 μg/ml Act-D, an RNA Synthesis Inhibitor, 30 min before *O. majorana* Treatment (350 µg/ml) for 24 h. Data are presented as mean ± SEM (n = 10) of three independent experiments. *P < 0.05, **P < 0.01, and ***P < 0.001 compared with controls; *P < 0.05 compared with the same treatment without Act-D

Figure 9, the outcomes reveal that Act-D cell pre-treatment did not modify the constitutive expression of Caspase-7 mRNA in any way when compared with untreated cells. However, the Caspase-7 mRNA was suppressed by the *O. majorana* aqueous extract via the most noteworthy effective doses of 150, 200 and 350 µg/m. This outcome proposes that *O. majorana* aqueous extract increases the Caspase-7 mRNA level by inducing it via the de Novo RNA synthesis.

**Table 1:** Description of the Plant under the Study

Parameter	Majorana
Botanical name	*Origanum majorana*
Family	*Lamiaceae* – mints, menthes
English name	knotted marjoram; marjoram
Vernacular name (in Arabian region, KSA)	Bardaqoosh, Wezzab, Doosh
Part used	The aerial parts
Checked names on Plant list http://www.theplantlist.org;	http://www.theplantlist.org/tpl1.1/search?q=Origanum Majorana

**Table 2:** Real-Time PCR Reactions Primers Sequences

Gene	Reverse primer	Forward primer
*CASPASE-7*	CTACCGCCGTGGGAACGATGGCAGA	CGAAGGCCCATACCTGTCACTTTATC
*p53*	GGGAGAGGAGCTGGTGTTG	GCCCCCAGGGAGCACTA
*BCl2*	GCCGGTTCAGGTACTCAGTCA	CATGTGTGTGGAGAGCGTCAA
*DR4*	GTGCTGTCCCATGGAGGTA	AGTACATCTAGGTGCGTTCCTG
*HO-1*	TGTTGCGCTCAATCTCCTCCT	ATGGCCTCCCTGTACCACATC
*NQO1*	CGTTTCTTCCATCCTTCCAGG	CGCAGACCTTGTGATATTCCAG
*GAPDH*	GGCATAGAGGTCTTTACGGATGTC	TATTGGCAACGAGCGGTTCC

**Table 3:** Phytochemical Screening of *O. majorana* Aqueous Extract Profile

Phytochemical	Extract content
Essential oils	+
Anthocyanins	+
Anthraquinones	+
Tannins	+
Glycosides	+
Alkaloids	+
Flavonoids	+
Phenols	+
Gums and mucilage	+
Terpenoids	-
Saponins	+
Steroids	+

**Table 4:** Total Flavonoids and Phenolic Contents in *O. majorana *Aqueous Extract Profile

Test	Concentration (mg equivalent/g DW)
Total phenolic content (TPC)	58.24 ± 1.82
Total flavonoids content (TFC)	75.31 ± 1.14

## Discussion

This study revealed that aqueous extracts of *O. majorana* are capable of inducing apoptosis in human breast cancer cells. Presumably, *O. majorana* aqueous extract content contained higher TFC and TPC (58.24 and 35.31 mg equivalent/g DW, respectively), which exceeded the concentration recently described by Bhardwaj and Dubey (2019) and Udaya et al., (2019), who found TFC and TPC of approximately 48 and 58 mg equivalent/g DW, respectively. Therefore, this extract method increased the quantitative profile of these compounds (TFC and TPC). In addition, the presence of gum and mucilage in the aqueous extract of *O. majorana* in this study was not recorded in (Chandra and Gonzalez, 2004), which could return to the efficiency of the cold extraction method at (0°C/6 h).

We found that cell viability was significantly inhibited by aqueous extracts of *O. majorana* (150, 200, and 350 μg/ml) after 24 and 48 h of culture. The dose-dependent, growth-inhibitory effect of the aqueous extract that was extracted at low temperature (0°C) differed significantly from that extracted at high temperature, ethanol and methanol solvents (Cipak et al., 2006; Erenler et al., 2016; Leeja and Thoppil, 2007; Makrane et al., 2018; Richter and Schellenberg, 2007; Singh et al., 2020). 

Notably, high ROS levels in mitochondria can result in free radicals attacking membrane phospholipids that go before mitochondrial film depolarisation. Mitochondrial depolarization, which is considered as an irreversible advance in apoptosis, triggers a cascade of caspases (Alkhateeb et al., 2021; Stohs and Bagchi). In addition, numerous breast cancer cells were vacuolated after treatment with aqueous extracts of *O. majorana*, which could induce cell death by apoptosis in MCF7 cells, as evidenced by mitochondrial activity, glucose uptake inhibition, expanded the activity of caspase-7, apoptotic genes, and oxidative stress, intracellular ROS accumulation (Baranauskaite et al., 2017). The improvement of ROS creation prompted expanded apoptosis occasions (Figure 2). Provided that mitochondrial morphology affects energy, imbalances and is endlessly modified by fission and fusion events, tightly coordinated activity between organelles and within mitochondrial dynamics are vital. Mitochondrial fusion results in impaired insulin-dependent glucose uptake (Erenler et al., 2016; Hsu et al., 2015; Makrane et al., 2018).

Apoptosis is a tightly regulated process and is heavily influenced by a few flagging pathways, such as mitochondrial pathways and caspases (Attoub et al., 2018). Caspase-7 is a particularly significant proapoptotic protein as it is involved in intrinsic and extrinsic apoptotic pathways (Athamneh et al., 2020; Attoub et al., 2018; Lowe and Lin, 2000). Caspase-7 activation is central to the induction of apoptosis, which requires the activation of initiator caspases (e.g., caspase-9 or -8) (Athamneh et al., 2020; Benhalilou et al., 2019; Lowe and Lin, 2000). Apoptosis induction with ROS production by malignant growth chemoprotective agents, such as doxorubicin (Tsang et al., 2003), incites disease cell passing as well as purposes DNA damage, leading to genomic instability (Baranauskaite et al., 2017; Gad et al., 2020).

On the other hand, caspase-7 mRNA induction by *O. majorana* aqueous extract treatment at the most noteworthy effective doses (150, 200, and 350 µg/ml) was completely suppressed by Act-D, suggesting that *O. majorana* upregulates caspase-7 mRNA expression through increased de novo RNA synthesis. 

Nevertheless, a large portion of these chemoprotective treatments against malignant growth are cytotoxic. Thus, the generation of new chemoprotective capable of inhibiting cell expansion (Elbekai et al., 2004; Grbović et al., 2013) and activating apoptosis in malignant growth cells are needed; however, with less or no reactions is significant and foreseen. 

Along this selected cell lines, to display the in vivo circumstance, our study used a human breast malignancy MCF7 cell line to predict human physiological reactions to exposure to *O. majorana* by researching the limit of this extract to hinder MCF7 cell development and expansion and to investigate the role of apoptosis in the effects of *O. majorana* treatment.

In addition, we have assessed the potential anticancer properties of *O. majorana* aqueous extract containing high concentrations of flavonoids and phenolic substances; previous research employed hot water as an aqueous solvent or alcoholic solvents such as methanol or ethanol (Bhardwaj and Dubey, 2019; Makrane et al., 2018; Udaya et al., 2019).

In conclusion, our findings showed that the aqueous extract of *O. majorana* collected in Abha, Saudi Arabia and extracted at low temperature (0°C/6 h) resulted in high flavonoid and phenolic content bearing anticancer and antioxidant properties. In addition, the dose-dependent growth inhibitory effect of the aqueous extract at the cooling temperature was higher than at the heating temperatures and attested to its potential as an effective anticancer agent.

## Author Contribution Statement

AG participated in the design of the study, participated in the practical work of the study, collecting the data and reviewed the manuscript. QA participated in the coordination of the study and reviewed the manuscript. WRA participated in the coordination of the study and reviewed the manuscript. AMA performed the statistical analysis and reviewed the manuscript. FGhA participated in the practical work of the study and reviewed the manuscript. ISA participated in the design of the study and reviewed the manuscript. GA participated in collecting the data and reviewed the manuscript. ASA participated in the design of the study, acquisition, participated in the practical work of the study, collecting the data and reviewed the manuscript. WSA carried out the design of the study, wrote, revised, edited the manuscript text and corresponding author for this study. All authors made a significant contribution to the work reported, read, revised, and agreed the final manuscript before submission.
